# A Randomized Controlled Study of Neurofeedback for Chronic PTSD

**DOI:** 10.1371/journal.pone.0166752

**Published:** 2016-12-16

**Authors:** Bessel A. van der Kolk, Hilary Hodgdon, Mark Gapen, Regina Musicaro, Michael K. Suvak, Ed Hamlin, Joseph Spinazzola

**Affiliations:** 1 Trauma Center at Justice Resource Institute (JRI), Brookline MA, United States of America; 2 Boston University School of Medicine, Department of Psychiatry, Boston, MA, United States of America; 3 National Child Traumatic Stress Network, Brookline, Massachusetts, United States of America; 4 Department of Psychology, Suffolk University, Boston MA, United States of America; 5 University or North Carolina School of Medicine, Department of Psychiatry, Chapel Hill, NC; 6 Western Carolina University, Psychology Department, Cullowhee, NC, United States of America; National Cancer Center, JAPAN

## Abstract

**Introduction:**

Brain/Computer Interaction (BCI) devices are designed to alter neural signals and, thereby, mental activity. This study was a randomized, waitlist (TAU) controlled trial of a BCI, EEG neurofeedback training (NF), in patients with chronic PTSD to explore the capacity of NF to reduce PTSD symptoms and increase affect regulation capacities.

**Study Design:**

52 individuals with chronic PTSD were randomized to either NF (n = 28) or waitlist (WL) (n = 24). They completed four evaluations, at baseline (T1), after week 6 (T2), at post-treatment (T3), and at one month follow up (T4). Assessment measures were:1. Traumatic Events Screening Inventory (T1); 2. the Clinician Administered PTSD Scale (CAPS; T1, T3, T4); 3. the Davidson Trauma Scale (DTS; T1-T4) and 4. the Inventory of Altered Self-Capacities (IASC; T1-T4). NF training occurred two times per week for 12 weeks and involved a sequential placement with T4 as the active site, P4 as the reference site.

**Results:**

Participants had experienced an average of 9.29 (*SD* = 2.90) different traumatic events. Post-treatment a significantly smaller proportion of NF (6/22, 27.3%) met criteria for PTSD than the WL condition (15/22, 68.2%), *χ2* (*n* = 44, *df* = 1) = 7.38, *p* = .007. There was a significant treatment condition x time interaction (*b* = -10.45, *t* = -5.10, *p*< .001). Measures of tension reduction activities, affect dysregulation, and affect instability exhibited a significant Time x Condition interaction. The effect sizes of NF (*d* = -2.33 within, d = - 1.71 between groups) are comparable to those reported for the most effective evidence based treatments for PTSD.

**Discussion:**

Compared with the control group NF produced significant PTSD symptom improvement in individuals with chronic PTSD, as well as in affect regulation capacities. NF deserves further investigation for its potential to ameliorate PTSD and to improve affect regulation, and to clarify its mechanisms of action.

## Scientific background and rationale

The potential of brain-computer interaction devices (BCI) to alter neural signals and associated mental activity makes them strong candidates to emerge as a new generation of psychiatric interventions [1]. BCIs utilize fMRI or EEG as information about brain activity and provide visual and/or auditory feedback to alter neural activity. Thus far, most BCI research has focused on helping physically disabled users communicate commands, such as stimulation of muscles in paralyzed individuals and in stroke rehabilitation [2].

However, a wide variety of BCIs are currently being explored for performance enhancement, mental focus and tranquility [3]. EEG neurofeedback (NF) training represents one of the earliest applications of BCIs, and even though it has been in use for about three decades with well documented effects in over 2000 peer reviewed scientific publications, serious questions remain about its clinical utility and the validity and scientific rigor of extant research [4].

NF is thought to indirectly modify behavior by changing neuronal activation or connectivity patterns in the CNS via operant conditioning. NF has been shown to be able to reshape neural activity, as measured by electroencephalogram (EEG) frequency components [5–7] and fMRI [8–11]. NF research has focused mainly on performance enhancement (e.g. [12]) and on clinical conditions such as Attention Deficit Hyperactivity Disorder (ADHD)[13–16]. A few studies have investigated its potential in the treatment of depression[17,18], substance abuse[19], and posttraumatic stress disorder (PTSD)[20–22].

In NF training, neural activity is recorded from scalp electrodes and fed back in real time to subjects in a readily understood, visual format (simple computer games). NF associated EEG changes have been correlated with changes in various functional outcomes, including cortico-motor excitability, memory, cognition, sleep, and mood, as well as increases in affect regulation and executive function, sustained attention, and working memory [23–25].

### Specific objectives

NF training may help individuals with PTSD acquire self-regulation skills by stabilizing EEG activity, and thereby improve focus and attention. The objective of this study was to investigate whether NF could substantially alter affect regulation capacities, and thereby improve PTSD symptomatology. This is particularly important because recovery from PTSD depends on being able to manage intense arousal [26]. Impaired affect regulation is a major cause of discontinuation of exposure-based PTSD treatments [27–29]. Improving affect regulation has been shown to 1) reduce the severity of PTSD symptoms, 2) decrease risk behaviors (e.g. suicidal and self-injurious behaviors, substance use) and 3) make subsequent exposure therapy more effective [30,31]. EEG markers of PTSD disordered arousal include increased cortical activation (manifested in reduced alpha activity) and increased theta/alpha ratio [32–34]. Brain activity in the alpha-1 band has been linked to attentional processes [35,36], while theta power has been linked to working memory performance [37,38], both of which are impaired in PTSD [26]. A recent study examining potential mechanisms for NF’s effects on PTSD found alterations in arousal (i.e., an increased sense of calm) correlated with changes in intrinsic network connectivity and alpha oscillations [21].

## Study Design

This randomized, waitlist-controlled trial evaluated the efficacy of NF to increase affect regulation and reduce PTSD symptoms in adults with multiple trauma exposures and treatment resistant PTSD (i.e., having received six or more months of trauma focused therapy without sustained self-reported clinical improvement).

### Study Sample

Following IRB review by the Justice Resource Institute Institutional Review Board and specific approval by that IRB of all aspects of the study, adults 18–58 years old with treatment non-responsive PTSD were recruited via newspaper and radio ads, the Trauma Center website (www.traumacenter.org), and solicitation from mental health professionals. The study was conducted between July 1, 2012 and July 1, 2015. Eligible subjects signed the IRB approved consent form that spelled out procedures, risks and benefits of the study. Trauma history was obtained by self-report ad scored on the Traumatic Events Screening Inventory [39], an 18-item self-report measure assessing lifetime occurrence of both acute (e.g. accident, natural disaster, loss) and interpersonal (e.g. neglect, separation, physical / sexual / emotional abuse, domestic violence) forms of trauma. Individuals were eligible if they met DSM-IV criteria for PTSD per the Clinician Administered PTSD Scale (CAPS) [40], and had received weekly trauma-focused psychotherapy for a minimum of six months. After completion of all initial evaluations subjects were randomly assigned by a computer generated randomization program to either 12 weeks of twice weekly NF or a waitlist (WL) control condition. Both groups were required to continue all ongoing treatments (psychotherapeutic and pharmacological) and to refrain from making changes in their current treatment regimens for the duration of this study. Participants in the WL condition were provided 24 NF sessions free of charge after the time 3 (week 12) follow-up evaluation.

Exclusion criteria included: unstable medical condition; receiving disability benefits; active suicide risk or life-threatening self-mutilation; psychotic or bipolar disorder; traumatic brain injury (TBI); history of seizures; current substance or alcohol abuse; ongoing traumatic exposure (such as domestic violence); changing ongoing treatment during the course of the study; Global Assessment of Functioning (GAF) score <40.

Of the 52 subjects in the ITT sample, 26 (50%) were on psychotropic mediation 21 (40%) were not, and 5 (10%) had missing data. A breakdown of medication rates and types for each group were as follows: waitlist control; 10 subjects on medication (41.7% of waitlist group) with 6 subjects (25%) on SSRIs, 3 subjects (12.5%) on benzodiazepines, 2 subjects (8.3%) on antianxiety, 2 subjects (8.3%) on buproprion, 2 subjects (8.3%) on an SSNRI, and 1 subject (4.2%) on a tricyclic antidepressant. Active NFB; 16 subjects on medication (57.1% of NF group), with 7 subjects (25%) on SSRIs, 4 subjects (14.3%) on stimulants, 3 subjects (10.7%) on antipsychotics, 5 subjects (17.8%) on benzodiazepines, and 3 subjects (10.7%) on buproprion.

### Evaluation Procedures

All data were collected and analyzed at the Trauma Center @ JRI. After providing informed consent participants completed four evaluations assessing psychological and behavioral functioning: at study baseline, at week 6 (i.e., session 12 of NF if in active treatment condition), 12 weeks (session 24 -post-treatment) and week 16 (one month follow up). The participants were compensated $25 for baseline and week 6 evaluations, $35 for week 12 evaluations, and $50 for 1-month follow-ups, totaling $135 for NF participants and $245 for WL participants who completed all evaluations. Evaluators were post-doctoral and master’s level clinicians who received training and ongoing supervision in administration of study measures. Inter-rater reliability on the CAPS was established at 80% agreement. Strenuous efforts were made to keep evaluators blind to treatment condition, though in one case the blinding was inadvertently compromised.

### NF Intervention Procedures

The NF system utilized EEGer neurofeedback software manufactured by EEG Spectrum International Education and Research, Inc. The system utilized a Procomp2 amplifier manufactured by Thought Technology LTD. The lowpass filtering is provided by the EEGer software. The training sites were fixed for all participants, and involved a sequential placement with T4 as the active site, P4 as the reference site, and the left ear (A1) as the ground (consistent with previous research that demonstrates increased R temporal lobe activation in PTSD)[41,42].

Training was intended to teach subjects to alter the power spectrum of certain filtered frequencies of activity; specifically, we sought to help subjects decrease the power spectrum of slow (2–6 Hz) and fast (22–36 Hz) activity while simultaneously increasing the power spectrum of mid-range (10–13 Hz starting point) activity. By convention, the slow activity we sought to decrease spans the delta and theta range and is generally associated with drowsiness and sleep, while the fast activity is called “high beta” and is associated with high levels of mental activation [43]. In contrast, we sought to help subjects enhance alpha activity, which is generally associated with a calm, relaxed state.

The training protocol employed standard inhibit frequencies of 2–6 HZ for slow activity and 22–36 HZ for fast EEG activity and a beginning reward frequency of 10–13 HZ. These spectral bands were selected based on previous research in studies of NF for PTSD [32,33], including the results of our pilot study [22]. Subjects completed a short checklist after every session, our internally generated “Checklist of Changes Observed After Neurofeedback Training”. Adjustment of the reward band was based on subjects’ responses to the questions on that checklist, and followed a flexible, principle-based manual that provided rules for adjusting the training protocol.

### NF training process

NF subjects had 24 training sessions, twice weekly, each lasting up to 30 minutes. Electrodes were applied; impedance was measured for each electrode and maintained below 10 kOhms. After initiating the EEG measurement, subjects were asked to relax and sit quietly while a baseline signal was obtained. Once a stable baseline signal was obtained, thresholds were set such that the 2–6 Hz activity was over the threshold 35% of the time, 10–13 Hz (or adjusted band, based on response) was over the threshold 65% of the time, and 22–36 Hz activity was over the threshold 25% of the time. Subjects received auditory and visual feedback indicating reward; specifically, auditory tones and progress in simple computer games, such as Packman or Space Race. Feedback “rewards” (positive progress in the visual feedback videogame and tones) were given every two seconds that the amplitude or magnitude of EEG activity in both of the inhibit frequency bands fell below the target threshold and the amplitude or magnitude of EEG activity in the reward band exceeded the target threshold. Further adjustment of the thresholds using the same parameters as above was made after approximately three minutes of active training. Any other adjustments to the thresholds were limited but based on clinical judgment. Finally, an artifact filter on the raw EEG was tailored for each subject in an effort to remove and minimize artifact during the training (EMG, EOG, blinking, etc.). No changes were made to the protocol except adjustments to the reward band frequency. These were made based on rated symptoms of over-arousal (including nightmares; sleep difficulties; hyperactivity; aggressive behavior, anger, anxiety; and self-reports of high arousal including self-harm, suicidal and/or homicidal ideation), and symptoms of under-arousal (including inattention, decreased alertness or mental clarity; nausea; depressive symptoms; and decreased energy/fatigue) captured by the *Checklist for Changes After Neurofeedback*, as well as clinical judgment. If participants reported significant over-arousal symptoms for at least two training sessions, the reward frequency was lowered by 1 Hz. This procedure was continued until the participant reported no change, positive benefit, or symptoms of under-arousal. If the participant reported symptoms of under-arousal, the reward band was raised by ½ Hz until those symptoms remitted. Training time started at 12 minutes; training time was raised in three-minute increments participants reported a positive change. All but one participant achieved an endstate of thirty minutes per NF session.

### Clinician Supervision, Fidelity, Assessment and Monitoring

NF sessions were conducted by experienced NF clinicians. Clinicians completed a session fidelity checklist designed to mirror the specific components of each session, requiring the rating of the full, partial or unsuccessful implementation of each component, any factors that impeded protocol adherence, and any modifications to protocol required. Changes to the starting protocol, such as frequency adjustments, were automatically recorded by the NFB software. Clinicians met weekly with the supervisor to review specifics of each NFB session and individual subject logs, session fidelity checklists, and protocol adjustments. Twenty percent of sessions were randomly selected for neurofeedback protocol review to assure that they matched adjustments dictated by supervisory staff.

## Measures

1. Clinician Administered PTSD Scale (CAPS) [40], a clinician administered interview that is considered the gold standard for assessing PTSD, was the primary outcome measure of the study. Each of the 17 DSM-IV-TR PTSD symptoms are assessed with regard to their frequency and intensity over the past month using a 5-point scale (0–4). Symptoms endorsed with a frequency equal to or greater than one, and an intensity of equal to or greater than two are considered to meet the minimum threshold to count as a symptom of PTSD. The CAPS can be scored to indicate whether an individual meets the DSM criteria for a PTSD diagnoses, and frequency and intensity items can be summed to produce a symptom severity rating that can range from 0–136, with severity score equal to or greater than 45 necessary for a PTSD diagnosis. The PTSD diagnoses variable was used to evaluate inclusion criteria, while the continuous PTSD severity score was the primary outcome for the study analyses.

2.The Davidson Trauma Scale (DTS) [44] is a self-report measure of PTSD assessing the severity and frequency of PTSD symptoms that is structured similarly to CAPS with participants rating the frequency and severity of the 17 DSM-IV-TR PTSD symptoms using a 0–4 scale. PTSD severity scores were computed by adding all of the frequency and intensity items of each symptom, which, like the CAPS, produces a continuous score that can range from 0–136.

3. Inventory of Altered Self-Capacities (IASC) [45] is a 63-item standardized self-report measure consisting of seven subscales that assess the following domains of self-related psychological problems: Interpersonal Conflicts, Idealization-Disillusionment, Abandonment Concerns, Identity Impairment, Susceptibility to Influence, Affect Dysregulation, and Tension Reduction Activities. The number of items per subscale ranges from 5–9 with each item rated using a 1 (never) to 4 (often) scale producing a continuous score for each subscale.

The CAPS, our primary outcome measure, was administered on three occasions (not mid-point, at week 6). We included the DTS as a secondary outcome measure because it is easier to administer; therefore, we could include it as part of the six-week assessment that occurred during NF-training (or the corresponding time on the waitlist). Including an additional assessment increases power to detect significant results in longitudinal studies. The IASC was administered as a secondary measure to evaluate whether NF impacted relevant emotion-regulation and interpersonal processes.

### Statistical Power

A post-hoc power analysis using a Monte Carlo simulation method [46] with the Mplus statistical software [47] was conducted to estimate obtained power. The Monte Carlo simulation method provides precise estimates of power for specific, hypothesized or evaluated, models and it can adjust for the missing data patterns in the observed data [48]. We conducted the power analysis using the estimates obtained from the multilevel growth curve models (GCMs, described below) using the CAPS and the DTS. These power analysis indicated power estimates of .56 and .77 to detect a d = .80 (usually considered the cutoff for large effect sizes) difference in change from pre-treatment to the follow-up assessment in the CAPS and DTS severity scores, respectively, between the two treatment conditions.

### Participants

**Fig 1:**
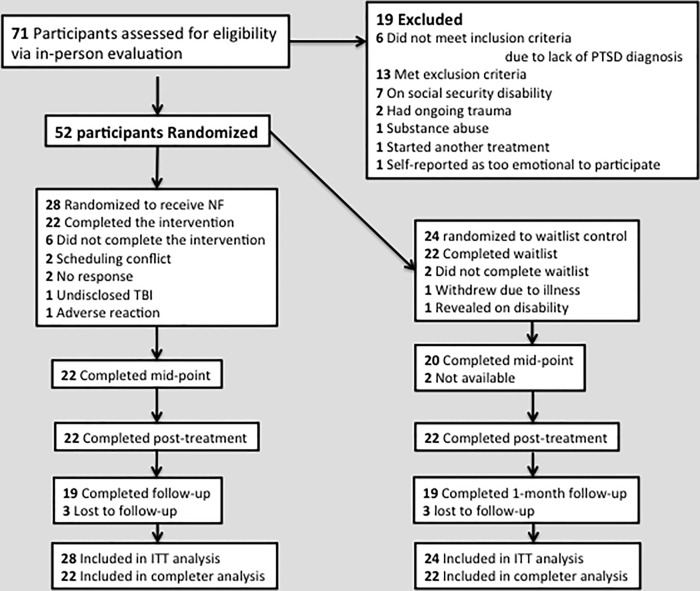
Flow of participants through the trial.

After initial phone screens 71 individuals were invited for a baseline assessment. Nineteen did not meet study criteria and were excluded: seven were on disability, six received subclinical scores on the CAPS, two due reported ongoing domestic abuse, one suffered a substance abuse relapse, one started another treatment, and one reported symptoms of psychosis. The remaining 52 individuals were randomized to either the WL (n = 24) or NF (n = 28) conditions and made up the intention-to-treat sample (ITT). Of the 28 individuals randomized to the NF group, six dropped out of treatment: four after having been randomized, but before starting the actual treatment (Fig 1). After starting NF two subjects dropped out—one had a previously undisclosed traumatic brain injury, and one subject reported increased flashbacks. Thus, 22 of the 28 participants assigned to NF completed the protocol. Of the 24 participants randomized to the WL group, one withdrew consent before treatment due to a medical illness; another was excluded after revealing that he received disability, leaving 22 participants assigned to the WL condition who completed the protocol. A chi-squared analysis showed that there was no significant difference between NF (6/28) and WL (2/24) ITT participants who failed to complete the protocol conditions, *χ2* (*n* = 52, df = 1) = 1.70, *p* = .192.

Table 1 presents demographic information for the entire sample and as a function of treatment condition. The sample was on average middle-aged and mostly white and female. A series of one-way ANOVAS and chi-squared tests revealed no statistically significant differences in demographic characteristics across conditions. A second series of one-way ANOVAs and chi-squared tests were then conducted to compare the treatment completers to ITT participants who dropped out or were excluded from the study. There was a significant difference for only one demographic variable, marital status, *χ2* (*n* = 48, df = 1) = 15.62, *p* = .004. Because drop-outs were relatively evenly distributed across conditions, we decided not to include marital status as a co-variate.

**Table 1:** Sample Demographic Information

	ITT Sample		
	Total	Waitlist	Neurofeedback
	(n = 52)	(n = 24)	(n = 28)
Age, mean (SD), y	44.40 (13.15)	42.45 (13.50)	46.04 (12.89)
Gender			
Female	42 (76.2)	17 (77.3)	25 (92.6)
Male	7 (23.8)	5 (22.7)	2 (7.4)
Ethnicity			
Black/African-American	4 (8.7)	2 (9.1)	2 (8.3)
Native American	1 (2.2)	1 (4.5)	0 (0.0)
White/Caucasian	35 (76.1)	15 (68.2)	20 (83.3)
Multi-Ethnic	4 (8.7)	3 (13.6)	1 (4.2)
Other	2 (4.3)	1 (4.5)	1 (4.2)
Marital Status			
Married	15(31.2)	8 (36.4%)	7 (26.9)
Separated	2 (4.2)	1 (4.5)	1 (3.8)
Divorced	6 (12.5)	2 (9.1%)	4 (15.4)
Single	**23** (47.9)	11 (50.0)	12 (46.2)
Widowed	2 (4.2)	0 (0.0)	2 (4.2)
Highest Level of Education			
Post-Graduate	7 (14.9)	4 (18.2)	4 (16.0)
Graduate	10 (21.3)	5 (22.7)	5 (20.0)
Some Graduate	8 (17.0)	4 (18.2)	4 (16.0)
College	10 (21.3)	4 (18.2)	6 (24.0)
Some College	11 (23.4)	5 (22.7)	6 (24.0)
High school graduate	1 (2.1)	0	1 (4.0)
Employment Status			
Full-Time	26 (54.2)	11 (50.0)	15 (57.7)
Part-Time	13 (27.1)	6 (27.3)	7 (26.9)
At Home Parent	2 (4.2)	1 (4.5)	1 (3.8)
Full-Time Student	2 (4.2)	1 (4.5)	1 (3.8)
Unemployed	5 (10.4)	3 (13.6)	2 (7.7)
Income			
$80,000 +	9 (19.6)	6 (28.6)	2 (8.0)
$60,000 - $79,000	3 (6.5)	4 (19.0)	5 (20.0)
$40,000 - $59,000	7 (15.2)	3 (14.3)	4 (16.0)
$26,000 - $39,000	7 (15.2)	2 (9.5)	5 (20.0)
$12,000 - $25,000	9 (19.6)	0 (0.0)	3 (12.0)
less than $12,000	8 (17.4)	5 (23.8)	4 (16.0)
Declined to answer	3 (6.5)	1 (4.8)	2 (8.0)

Note: Except for Age, for which mean and standard deviation is reported, n’s with percentages in parenthesis are reported.

## Data Analyses

Chi-square analyses were first were used to evaluate the impact of NF on PTSD diagnoses (present/absent). Next multilevel Growth Curve Modeling (GCM) using the mixed procedure of the Statistical Package for the Social Sciences (SPSS [48]) examined change in PTSD symptoms and other study variables across the course of treatment through one-month post treatment and whether these changes significantly varied across condition. Multilevel GCMs have become the standard for analyzing psychotherapy outcome data because of several advantages that this approach offers (i.e., efficiency in dealing with missing observations, efficient and powerful estimation techniques, and modeling flexibility [49]). This allowed us to include the entire intention-to-treat sample without using data imputation procedures. Time was modeled by including the number of weeks since baseline assessment (0, 6, 12, and 16, for pre-treatment, mid-treatment, post-treatment, and one-month post-treatment assessments). Prior to examining the impact of treatment condition on change, various unconditional change models (examining change without predictors) were evaluated to determine the most reliable manner to model time (e.g., linear using number of months, quadratic using number of months and number of months squared, or non-linear using a natural-log transformation of number of months). The best fitting change model was determined by examining the difference in the-2 Log Likelihood (i.e., deviance) estimate between competing models, which follows a chi-square distribution. To examine the impact of treatment condition on change in outcomes, a treatment condition dummy-coded variable was added as a predictor of change parameters (to test treatment condition x time interactions). Effect sizes (d) for differences in change between conditions was computed by the procedures described by Fiengold [50] producing effect size estimates comparable to those derived from more traditional repeated measures designs (e.g., repeated measures ANOVA).

### Subject Attrition and Missing Data

One of the benefits of using multilevel GCM is that we could use maximum likelihood estimation, so that subject attrition and missing data did not affect the analyses as they would for less nuanced methods. Data were screened for patterns of missingness as MLE does assume that data is missing at random.

## Results

On average participants endorsed exposure to 9.29 (*SD* = 2.90) of the 18 traumatic events assessed by the TESI, which did not significantly vary as a function of condition, *F* (1,43) = .02, *p* = .879. The most frequently endorsed events were childhood caregiver emotional abuse (78.8%), sexual abuse (69.2%) and domestic violence, 61.5%. At baseline, all participants met past month criteria for PTSD, and there was not a significant difference between WL (18/24, 75.0%) and NF conditions (24/27, 88.9%) meeting PTSD criteria during the past week, *χ2* (*n* = *51*, *df* = 1) = 1.69, *p* = .194. At week 12 (post-treatment) assessment, a higher proportion of WL participants (15/22, 68.2%) met criteria for PTSD than participants receiving NF (6/22, 27.3%), *χ2* (*n* = 44, *df* = 1) = 7.38, *p* = .007. At the week 16 (one-month post-treatment), a higher proportion of WL participants (17/19, 90%) met criteria for PTSD in the past month than participants receiving NF (8/19, 42%), *χ2* (*n* = 38, *df* = 1) = 9.47, *p* = .002.

**Fig 2:**
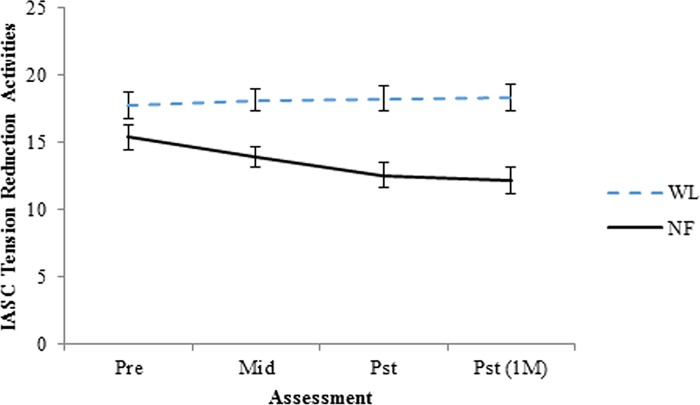
Change in PTSD symptom severity (Total CAPS score) as a function of treatment condition. WL = waitlist, NF = Neurofeedback. Standard Error bars included at each assessment.

CAPS severity scores were approximately normally distributed at each time point, with the highest skewness-to-standard-error of skewness ratio being1.91 at the post-assessment. The unconditional change model for the CAPS severity score indicated that modeling time as the natural-log transformation of number of weeks since baseline fit the data best accounting for 79.4% of the within-subjects variance. This pattern of change was characterized by large initial decreases in symptoms (during treatment) that flatten out over time (during the follow-up period). A significant treatment condition x time interaction emerged (*b* = -10.45, *t* = -5.10, *p*< .001). The nature of this effect is depicted in Fig 2 with change over time and associated effect size estimates reported in Table 2. Both the WL (*d* = -.62) and NF (*d* = -2.33) conditions exhibited significant decreases from the pre-treatment to the second (1 month) post treatment assessment; however, this decrease was substantially larger for the NF condition (*d* = -1.71). Both groups exhibited small decreases in CAPS severity score from the first to the second post-treatment to follow-up that did not significantly differ between conditions (*d* = -.16). The average decrease in CAPS score from the pre-treatment to the 1-month post treatment assessments was 40.35 for the NF condition and 10.78 for the WL group, the former well above and the latter well below the commonly adopted 20-point change in CAPS criteria used to indicate clinically significant change [49]. The bottom portion of Table 2 depicts the results when restricting the analyses to completers only and indicates that the results when using the ITT sample or the completers only sample were virtually identical.

**Table 2:** Pre-treatment, Post-treatment, and One-Month Post-treatment CAPS levels and Change Estimates

Sample		Pre		Pst		1 Month Post		ΔPre-1 Month Post	effect-size (d)
		n	*M* (*95%CI*)	n	*M* (*95%CI*)	n	*M* (*95%CI*)	*M* (*95%CI*)	Pre-Pst(1M)
ITT	WL	24	76.24 (69.13, 83.36)	22	66.49 (57.39, 75.6)	19	65.46 (55.83, 75.1)	-10.78 (-19.1, -2.48)	-0.62
N = 52	NF	28	79.45 (72.86, 86.04)	22	42.95 (34.1, 51.8)	19	39.1 (29.69, 48.51)	-40.35 (-48.67, -32.12)	-2.33
	Difference		3.2 (-6.5, 12.9)		-23.54 (-36.24, -10.85)		-26.36 (-39.83, -12.9)	-29.6 (-41.33, -17.87)	-1.71
Completers	WL	22	75.18 (67.84, 82.51)	22	65.68 (56.48, 74.88)	19	64.68 (54.96, 74.39)	-10.5 (-18.83, -2.19)	-0.61
N = 44	NF	22	80.98 (73.64, 88.31)	22	44.12 (34.92, 53.31)	19	40.23 (30.51, 49.95)	-40.75 (-49.11, -32.47)	-2.38
	Difference		5.8 (-4.57, 16.17)		-21.56 (-34.57, -8.55)		-24.45 (-38.19, -10.7)	-30.28 (-42.05, -18.51)	-1.77

Note: WL = waitlist condition, NF = Neurofeedback condition, Pre = pre-treatment assessment, Pst = immediate post-treatment assessment, Pst (1M) = 1-month post-treatment assessment; *M* = mean, *95%CI* = 95% Confidence Interval, *d* = effect size indicator with .2, .5, and .8 indicating small, medium, and large effect sizes.

Estimates for the means of the self-report measures administered at pre-treatment, mid-treatment, post-treatment, and at the follow-up assessments are displayed in Table 3 with the corresponding change parameters depicted in Table 4. DTS scores were also approximately normally distributed at each time point, with the highest skewness-to-standard error of skewness ration being 3.60 at the post-treatment assessment. A linear change model best fit the data for the DTS total score. Mirroring the CAPS severity score findings, a significant treatment condition x time interaction emerged for the DTS (*b* = -1.52, *t* = -3.89, *p*< .001). The WL condition did not exhibit a significant decrease in pre-post change in the DTS (*d* = -.25) while the NF condition exhibited a significant and large decrease (*d* = -1.23) with a large effect size difference between conditions (*d* = -.97). The analyses for the completer only sample, like for the CAPS analysis, was virtually identical. Therefore, we present the results for the self-report measures for only the ITT sample.

**Table 3:** Pre-treatment, Mid-Treatment, Post-treatment, and One-Month Post-treatment Means and Confidence Intervals for Self-Report Measures

Variable	Condition	Pre	Mid	Pst	Pst (1M)
		Est. (95%CI)	Est. (95%CI)	Est. (95%CI)	Est. (95%CI)
Davidson Trauma Scale (DTS)					
	WL	62.97 (52.47, 73.48)	60.59 (51.45, 69.74)	58.21 (49.26, 67.16)	56.62 (47.09, 66.15)
	NF	67.28 (57.55, 77.00)	55.74 (47.25, 64.22)	44.19 (35.76, 52.63)	36.5 (27.4, 45.6)
	Difference	4.30 (-10.01, 18.62)	-4.86 (-17.33, 7.62)	-14.02 (-26.32, -1.72)	-20.12 (-33.3, -6.95)
	Difference d	0.18	-0.23	-0.66	-0.89
IASC interpersonal conflicts					
	WL	23.49 (21.11, 25.87)	23.04 (20.87, 25.22)	22.6 (20.23, 24.97)	22.3 (19.61, 24.98)
	NF	19.22 (16.99, 21.45)	18.28 (16.21, 20.34)	17.33 (15.05, 19.62)	16.71 (14.09, 19.32)
	Difference	-4.27 (-7.53, -1.01)	-4.77 (-7.77, -1.77)	-5.26 (-8.56, -1.97)	-5.59 (-9.34, -1.84)
	Difference d	-0.76	-0.94	-0.96	-0.90
IASC tension reduction activities					
	WL	17.75 (15.86, 19.63)	18.14 (16.44, 19.83)	18.26 (16.4, 20.12)	18.31 (16.35, 20.27)
	NF	15.55 (13.78, 17.32)	13.25 (11.61, 14.89)	12.52 (10.70, 14.33)	12.20 (10.28, 14.12)
	Difference	-2.2 (-4.78, 0.39)	-4.89 (-7.25, -2.53)	-5.74 (-8.34, -3.14)	-6.11 (-8.85, -3.37)
	Difference d	-0.50	-1.25	-1.35	-1.36
IASC affect dysregulation total					
	WL	25.14 (22.11, 28.17)	24.63 (22.03, 27.23)	24.12 (21.46, 26.78)	23.78 (20.81, 26.75)
	NF	22.35 (19.5, 25.19)	20 (17.54, 22.46)	17.65 (15.08, 20.22)	16.08 (13.18, 18.99)
	Difference	-2.79 (-6.95, 1.36)	-4.63 (-8.21, -1.05)	-6.47 (-10.17, -2.77)	-7.7 (-11.85, -3.54)
	Difference d	-0.39	-0.76	-1.04	-1.13
IASC affect skill deficits					
	WL	13.68 (11.69, 15.66)	13.4 (11.72, 15.09)	13.13 (11.43, 14.83)	12.95 (11.07, 14.83)
	NF	12.18 (10.32, 14.04)	10.83 (9.24, 12.43)	9.49 (7.85, 11.13)	8.59 (6.76, 10.43)
	Difference	-1.5 (-4.22, 1.22)	-2.57 (-4.89, -0.25)	-3.64 (-6, -1.28)	-4.35 (-6.98, -1.72)
	Difference d	-0.33	-0.65	-0.94	-1.04
IASC affect instability					
	WL	11.45 (10.00, 12.90)	11.22 (9.95, 12.49)	10.99 (9.68, 12.3)	10.84 (9.38, 12.29)
	NF	10.15 (8.79, 11.51)	9.17 (7.97, 10.37)	8.18 (6.92, 9.45)	7.52 (6.10, 8.95)
	Difference	-1.3 (-3.29, 0.69)	-2.06 (-3.80, -0.31)	-2.81 (-4.63, -0.99)	-3.32 (-5.35, -1.28)
	Difference d	-0.38	-0.68	-0.90	-0.97
IASC identity impairement total					
	WL	23.16 (19.77, 26.54)	22.9 (19.88, 25.92)	22.65 (19.52, 25.79)	22.48 (19.02, 25.95)
	NF	21.40 (18.23, 24.58)	19.75 (16.9, 22.61)	18.1 (15.08, 21.13)	17.00 (13.62, 20.39)
	Difference	-1.75 (-6.39, 2.89)	-3.15 (-7.31, 1.01)	-4.55 (-8.91, -0.19)	-5.48 (-10.32, -0.64)
	Difference d	-0.22	-0.44	-0.62	-0.68
IASC idealization-disillusionment					
	WL	19.51 (16.53, 22.49)	19.44 (16.55, 22.33)	19.37 (16.25, 22.49)	19.32 (15.89, 22.75)
	NF	16.31 (13.58, 19.05)	15.12 (12.45, 17.79)	13.93 (10.99, 16.87)	13.13 (9.86, 16.40)
	Difference	-3.2 (-7.24, 0.84)	-4.32 (-8.25, -0.39)	-5.44 (-9.73, -1.15)	-6.19 (-10.93, -1.45)
	Difference d	-0.46	-0.65	-0.75	-0.77
IASC abandonment concerns					
	WL	21.5 (18.28, 24.73)	21.4 (18.45, 24.36)	21.37 (18.17, 24.58)	21.36 (18.01, 24.71)
	NF	20.25 (17.22, 23.28)	16.9 (14.06, 19.74)	15.83 (12.71, 18.95)	15.37 (12.09, 18.65)
	Difference	-1.26 (-5.68, 3.17)	-4.51 (-8.61, -0.40)	-5.54 (-10.01, -1.07)	-5.99 (-10.68, -1.30)
	Difference d	-0.17	-0.65	-0.73	-0.76
IASC susceptibility to influence					
	WL	19.08 (15.83, 22.34)	18.64 (15.78, 21.51)	18.2 (15.36, 21.04)	17.91 (14.87, 20.94)
	NF	17.79 (14.73, 20.84)	16.12 (13.41, 18.82)	14.44 (11.71, 17.17)	13.32 (10.37, 16.28)
	Difference	-1.29 (-5.76, 3.17)	-2.53 (-6.47, 1.42)	-3.76 (-7.7, 0.18)	-4.58 (-8.82, -0.35)
	Difference d	-0.17	-0.38	-0.56	-0.64

Note: WL = waitlist condition, NF = Neurofeedback condition, Pre = pre-treatment assessment, Pst = immediate post-treatment assessment, Mid = mid-treatment assessment, Pst (1M) = 1-month post-treatment assessment; *M* = mean, *95%CI* = 95% Confidence Interval, *d* = effect size indicator with .2, .5, and .8 indicating small, medium, and large effect sizes.

**Table 4:** Change Estimates for Self-Report Measures

Measures						
Variable		Linear/Ln Change	ΔPre-PST	effect-size (d)	ΔPST-FU	effect-size (d)
UCC Model	Condition	(per week)		Pre-Pst		Pst-FU
Con x Time		Est. (95%CI)	Est. (95%CI)		Est. (95%CI)	
Davidson Trauma Scale (DTS)						
Linear	WL	-0.4 (-0.97, 0.17)	-4.76 (-11.6, 2.07)	-0.19	-1.59 (-3.87, 0.69)	-0.06
Con x Time	NF	**-1.92 (-2.47, -1.37)**	**-23.08 (-29.68, -16.48)**	-0.92	**-7.69 (-9.89, -5.49)**	-0.31
*p*< .001	Difference	**-1.53 (-2.32, -0.74)**	**-18.32 (-27.82, -8.82)**	-0.73	**-6.11 (-9.27, -2.94)**	-0.24
	Difference d	-1.16	-0.73		-0.24	
IASC interpersonal conflicts						
Linear	WL	-0.07 (-0.23, 0.08)	-0.9 (-2.8, 1)	-0.15	-0.3 (-0.93, 0.33)	-0.05
Con x Time	NF	**-0.16 (-0.31, 0)**	**-1.89 (-3.74, -0.03)**	-0.32	**-0.63 (-1.25, -0.01)**	-0.11
*p* = .456	Difference	-0.08 (-0.3, 0.14)	-0.99 (-3.64, 1.67)	-0.17	-0.33 (-1.21, 0.56)	-0.06
	Difference d	-0.23	-0.17		-0.06	
IASC tension reduction activities						
Natural Log	WL	0.2 (-0.51, 0.91)	0.51 (-1.31, 2.32)	0.11	0.05 (-0.14, 0.24)	0.01
Con x Time	NF	**-1.18 (-1.87, -0.49)**	**-3.03 (-4.81, -1.26)**	-0.64	**-0.32 (-0.50, -0.13)**	-0.07
*p* = .007	Difference	**-1.38 (-2.37, -0.39)**	**-3.54 (-6.08, -1.00)**	-0.75	-0.37 (-0.64, -0.11)	-0.08
	Difference d	-0.86	-0.75		-0.08	
IASC affect dysregulation total						
Linear	WL	-0.09 (-0.28, 0.11)	-1.02 (-3.36, 1.32)	-0.15	-0.34 (-1.12, 0.44)	-0.05
Con x Time	NF	**-0.39 (-0.58, -0.2)**	**-4.7 (-6.97, -2.42)**	-0.67	**-1.57 (-2.32, -0.81)**	-0.22
*p* = .028	Difference	**-0.31 (-0.58, -0.03)**	**-3.68 (-6.94, -0.41)**	-0.53	**-1.23 (-2.31, -0.14)**	-0.18
	Difference d	-0.68	-0.53		-0.18	
IASC affect skill deficits						
Linear	WL	Est. (95%CI)	Est. (95%CI)		Est. (95%CI)	
Con x Time	NF	-0.05 (-0.17, 0.08)	-0.55 (-2.05, 0.96)	-0.12	-0.18 (-0.68, 0.32)	-0.04
*p* = .045	Difference	**-0.22 (-0.35, -0.10)**	**-2.69 (-4.15, -1.23)**	-0.59	**-0.9 (-1.38, -0.41)**	-0.20
	Difference d	**-0.18 (-0.35, -0.003)**	**-2.14 (-4.24, -0.05)**	-0.47	**-0.71 (-1.41, -0.02)**	-0.16
		-0.63	-0.47		-0.16	
IASC affect instability						
Linear	WL	-0.04 (-0.13, 0.05)	-0.46 (-1.56, 0.64)	-0.14	-0.15 (-0.52, 0.21)	-0.05
Con x Time	NF	**-0.16 (-0.25, -0.08)**	**-1.97 (-3.04, -0.90)**	-0.58	**-0.66 (-1.01, -0.30)**	-0.19
*p* = .053	Difference	-0.13 (-0.25, 0.001)	-1.51 (-3.04, 0.02)	-0.45	-0.5 (-1.01, 0.01)	-0.15
	Difference d	-0.60	-0.45		-0.15	
IASC identity impairment total						
Linear	WL	-0.04 (-0.25, 0.16)	-0.50 (-2.97, 1.96)	-0.06	-0.17 (-0.99, 0.65)	-0.02
Con x Time	NF	**-0.28 (-0.48, -0.07)**	**-3.30 (-5.72, -0.88)**	-0.41	**-1.10 (-1.91, -0.29)**	-0.14
*p* = .110	Difference	-0.23 (-0.52, 0.05)	-2.80 (-6.25, 0.66)	-0.35	-0.93 (-2.08, 0.22)	-0.12
	Difference d	-0.51	-0.35		-0.12	
IASC idealization-disillusionment						
Linear	WL	-0.01 (-0.18, 0.15)	-0.14 (-2.12, 1.83)	-0.02	-0.05 (-0.71, 0.61)	-0.01
Con x Time	NF	**-0.2 (-0.36, -0.04)**	**-2.39 (-4.32, -0.46)**	-0.35	**-0.8 (-1.44, -0.15)**	-0.12
*p* = .109	Difference	-0.19 (-0.42, 0.04)	-2.24 (-5.01, 0.52)	-0.33	-0.75 (-1.67, 0.17)	-0.11
	Difference d	-0.50	-0.33		-0.11	
IASC abandonment concerns						
Natural Log	WL	-0.05 (-1.19, 1.09)	-0.13 (-3.05, 2.79)	-0.02	-0.01 (-0.32, 0.29)	0.00
Con x Time	NF	-1.72 (-2.84, -0.60)	**-4.42 (-7.28, -1.55)**	-0.59	**-0.46 (-0.76, -0.16)**	-0.06
*p* = .041	Difference	-1.67 (-3.27, -0.07)	**-4.29 (-8.38, -0.19)**	-0.57	**-0.45 (-0.88, -0.02)**	-0.06
	Difference d	-0.62	-0.57		-0.06	
IASC susceptibility to influence						
Linear	WL	-0.07 (-0.25, 0.10)	-0.88 (-3.00, 1.23)	-0.12	-0.29 (-1.00, 0.41)	-0.04
Con x Time	NF	**-0.28 (-0.45, -0.11)**	**-3.35 (-5.41, -1.29)**	-0.44	**-1.12 (-1.80, -0.43)**	-0.15
*p* = .099	Difference	-0.21 (-0.45, 0.04)	-2.47 (-5.42, 0.49)	-0.33	-0.82 (-1.81, 0.16)	-0.11
	Difference d	-0.51	-0.33		-0.11	

Note: WL = waitlist condition, NF = Neurofeedback condition, *Est*. = estimate, *95%CI* = 95% Confidence Interval, *d* = effect size indicator with .2, .5, and .8 indicating small, medium, and large effect sizes, ΔPre-Pst(1M) = the estimate of total change from the pre-treatment to the 1-month post-treatment assessment, NA = Not Applicable (there is not quadratic estimate for linear and natural-log change models). The far left column denotes the outcome (underlined), the unconditional model (UCC) that best fit the data (linear, quadratic, or natural log), and the significance of the overall time x condition interaction (con x time). Z–because quadratic change models involve two change parameters (linear, quadratic) the 95% confidence intervals for these models could not be calculated.

As depicted in Table 4, four of the IASC subscales (tension reduction activities, affect dysregulation total, affect skill deficits, affect instability) exhibited significant Time x Condition interactions, with a fifth (affect instability) approaching statistical significance (*p* = .053). The interpersonal conflict and identity related subscales did not exhibit significant Time x Condition interactions. The IASC subscale with the largest difference between the two conditions was tension reduction, with the NF group exhibiting significant decreases and the WL group exhibiting slight increases. The effect size for the difference in tension reduction activities from pre-treatment to the post-treatment assessment was -.75.

## Discussion

Twenty-four sessions of NF produced significant improvements in PTSD symptomatology in multiply traumatized individuals with PTSD who had not responded to at least six months of trauma-focused psychotherapy, compared to a waitlist control group that continued to receive treatment as usual. The effect sizes of NF in this study (*d* = -2.33 within, d = - 1.71 between groups) is comparable to the results reported for the best evidence based treatments for PTSD: Prolonged Exposure, CBT and EMDR, which, like this study, also generally have employed TAU control groups, and better than any published drug intervention for PTSD [51]. The rate of completion of the NF protocol (79%) was comparable to reported exposure-based PTSD treatments (76%) [52]. In this study 72.7% of the NF sample no longer met criteria for PTSD. This is comparable to the 62% reported in metanalyses of other treatment studies [53]. Only one participant in the active treatment condition (4%) reported significant side effects, an increase in flashbacks.

The NF subjects also had statistically significant improvements in measures of affect regulation, identity impairment, abandonment concerns, and tension reduction activities. In contrast with most evidence based therapies for PTSD, which focus on processing memories of traumatic events, the target of NF is neural regulation and stabilization. Since lack of self-regulation has been identified as a principal cause of failure of exposure-based treatments [27–30], NF may be particularly helpful for traumatized individuals who are too anxious, dissociated or dysregulated to tolerate exposure based treatments. Finding cost-effective treatments for PTSD and other psychiatric conditions is particularly important in light of the limitations of existing treatments. Our results suggest that NF deserves further investigation for its potential to improve affect regulation, executive functioning and attention.

The former Director of the US National Institutes of Mental Health, calling for the development of a next generation of interventions, has noted that four decades of drug development has resulted in over 20 antipsychotics and over 30 antidepressants that have not demonstrably reduced the morbidity or mortality of mental disorders [54]. In an emerging new framework mental disorders are considered to be driven, at least in part, by abnormalities in underlying neural circuits [55]. A concerted effort is currently underway to map these networks, the so-called “human connectome project” [56,57]. The fact that mental disorders frequently are associated with abnormal brain-wave patterns and neural connectivity, including those measured by the EEG, lends support this approach [58].

Neurofeedback is a promising change agent for habitual dysfunctional neuronal patterns. The vast potential of EEG. based brain-computer interface techniques to train neuronal patterns is illustrated by recent research at the University of Minnesota. Using advanced functional neuroimaging including BOLD functional fMRI and EEG source imaging, they trained normal research subjects to control the flight of a virtual helicopter in 3-dimensional space through an obstacle course, based only upon motor imagination [59]. The equipment used in our study was much less sophisticated than that used in the helicopter navigation study, but it cost less than $10,000. If further research confirms the results from our study, neurofeedback has the potential of becoming widely available in community settings since can be economically administered by well-trained technicians in small offices and clinics.

### Limitations

There are major limitations in this relatively small, non-placebo controlled study, including: 1) This study employed a waitlist control group that received TAU (psychotherapy + medications). While this is an appropriate control, findings would be more robust with a sham control condition; 2) This study only had a one-month follow up. Further studies are needed to establish the relative permanency of NF generated clinical improvements and investigate the necessity for follow-up booster sessions. 3) In this study we used clinical indications- PTSD symptoms- to guide our approach. It remains to be determined whether it is optimal to target specific abnormalities in brain EEG patterns, or clinical symptomatology in NF research. 4) This study did not examine to what degree clinical changes are correlated with specific alterations in EEG, or other neural activation patterns. 5) The NF protocol targeted R temporal lobe EEG patterns. Future research need to determine the optimal targets and procedures for the treatment of PTSD and other clinical conditions, including whether there are optimal protocols for different clinical conditions, or whether psychiatric patients are best served by individualized targeted interventions that use advanced EEG imaging technologies such as sLORETA [60].

Finally, the power analysis indicated that the study was slightly underpowered. There are two primary problems with underpowered studies: 1) increased risk of Type II errors, and 2) insufficient sample to produce unbiased or stable parameter estimates. Regarding #1, the current study produced hypothesized statistically significant effects ruling out Type II error. Regarding #2, the Monte Carlo procedure used for the power analysis produced estimates of 0.1%, 4.8%, and 92.6% for parameter estimate bias, standard error bias, and coverage, respectively, for the condition x time interaction for the CAPS, and 0.04%, 5.05,% and 93.5% for the DTS power. As per criteria specified by Muthén and Muthén [46] these values suggest that the sample size for the current study produced unbiased and stable estimates. The current study produced statistically significant effects despite being underpowered, which mitigates concerns that have been expressed regarding the use of post-hoc power analysis to argue that the results of negative trials may be meaningful despite the failure to detect significant effects due to being underpowered (e.g., [61]). The current study used post-hoc analysis to investigate the stability of the produced coefficients, and we followed recommendations of those who caution against the use of post-hoc test to report confidence intervals for coefficients.

### Future directions

This study suggests that BCIs may have similar clinical potential for psychiatry as they do for rehabilitation medicine. Further clinical trials are needed to further substantiate to what degree BCIs can improve attention and affect regulation, and enhance cognitive performance and executive functioning. Using a combination of fMRI and quantitative EEG technology to define neural circuitry abnormalities, and studying the capacity of targeted neurofeedback interventions to alter these circuits would be a major step in that direction (e.g.11). Clarifying to what degree NF induced psychological changes are correlated with specific changes in neural activity will be a complex scientific challenge akin to correlating the clinical effects of various psychiatric medications with specific neurochemical changes in the brain.

## Supporting Information

S1 File CONSORT Checklist Part 1 (PDF)

S2 File CONSORT Checklist Part 2 (PDF)

S3 File Study Protocol (PDF)
