# Correction: A Randomized Controlled Study of Neurofeedback for Chronic PTSD

**DOI:** 10.1371/journal.pone.0215940

**Published:** 2019-04-24

**Authors:** Bessel A. van der Kolk, Hilary Hodgdon, Mark Gapen, Regina Musicaro, Michael K. Suvak, Ed Hamlin, Joseph Spinazzola

The graph shown in Fig 2 is incorrect. Please see the correct Fig 2 here.

**Fig 2:**
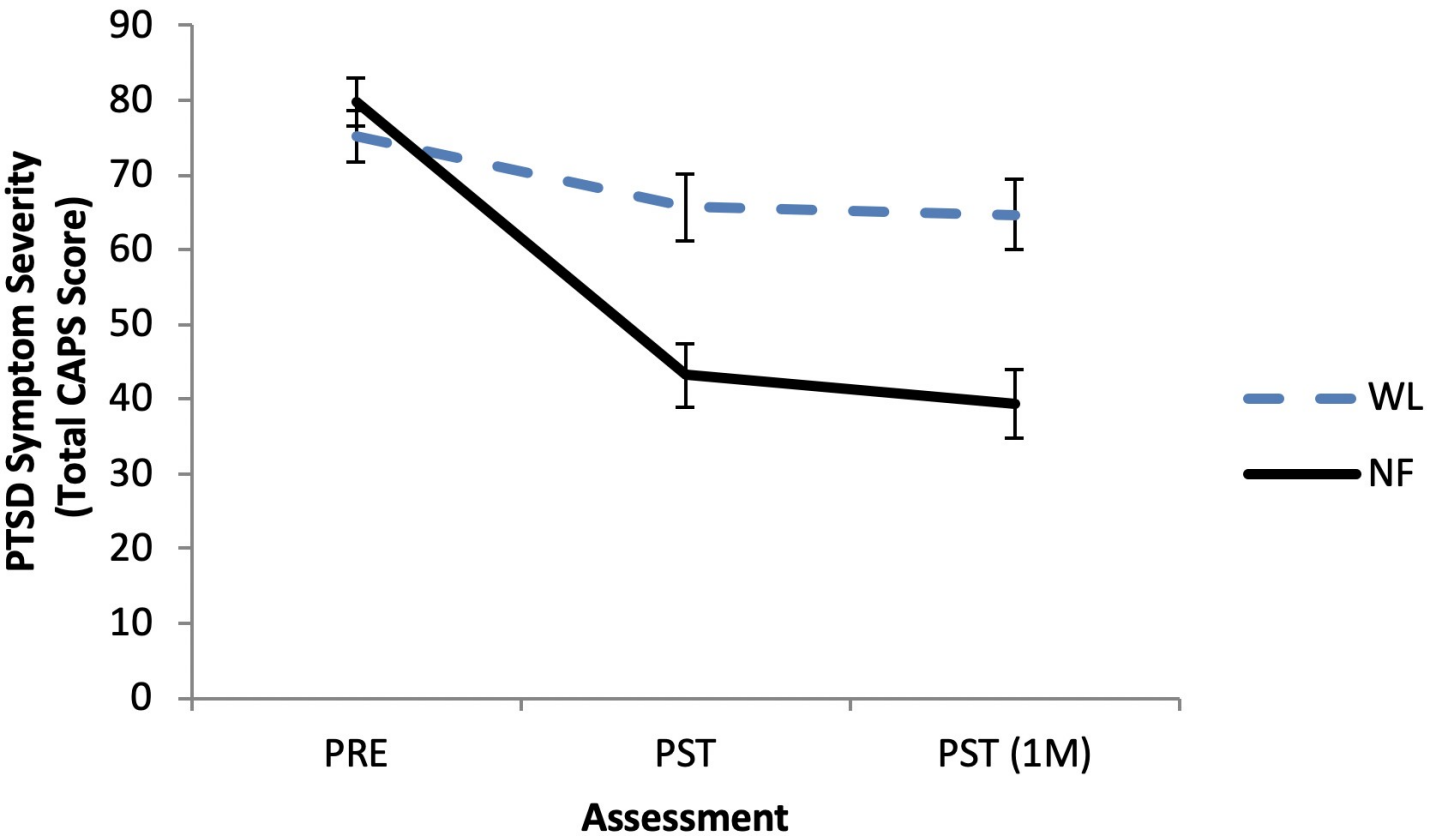
Change in PTSD symptom severity (Total CAPS score) as a function of treatment condition. WL = waitlist, NF = Neurofeedback. Standard Error bars included at each assessment.
