# Supplementary material for: A Randomized Controlled Study of Neurofeedback for Chronic PTSD
Source: PLoS One. 2016 Dec 16;11(12):e0166752. doi: 10.1371/journal.pone.0166752 (PMC5161315; doi:10.1371/journal.pone.0166752)
Supplement: S3 File — (PDF) [file pone.0166752.s003.pdf]

Justice Resource Institute
Institutional Review Board
Protocol Changes to Research
2008-06

1. **Date of this submission or revision**: 12/09/2011
2. **Title of Study**: Application of Neurofeedback as a Mechanism of Affect Regulation Treatment of Adults with Complex Adaptation to Chronic Interpersonal Trauma Exposure
3. **JRI IRB Code Number Assigned** (*contact IRB Chair)*: 2008-006
4. **Principal Investigator Information:**
   Name/Degree: Bessel A. van der Kolk, M.D.
   Address: 1269 Beacon Street, Brookline MA 02446
   Phone: 617 232 1303 x 206
   Email: besselvanderkolk@gmail.com
   License: Medical – Psychiatry specialization
   Affiliation: Trauma Center at JRI

   Names/Titles of Members of Research Team:

   Bessel van der Kolk, M.D., Project Director & Principal Investigator.
   Daphne Davis, Ph.D., Project Coordinator.
   Richard Jacobs, Psy.D., Project Supervisor.
   Ed Hamlin, Ph.D., Project Supervisor.
   Hilary Hodgdon, Ph.D., Interventionist.
   Regina Musicaro, B. A., Interventionist, Study Technician.

5. **Location(s) of Study:** Trauma Center at 1269 Beacon St., Brookline, MA 02446

6. **Starting Date and expected total duration of study:** 12/12/2011-9/1/2012

7. **Number of subjects to be studied:** Our aim is to enroll 40 individuals with 20 assigned to the active treatment group and 20 assigned to a waitlist control group.

8. **Relevant characteristics of subjects:** (*gender, age, ethnic/socioeconomic background; if individuals served by JRI (current or former) or their relatives, or if employees of JRI, specify which programs)*
   The initial year included 23 individuals with 20 enrolled in the study. Of those 20, 3 were men and 17 were women ranging in ages from 27-59. During year two, we enrolled 24 out of 36 individuals screened (16 females, and 8 males between the ages of 32 and 62). 16 completed the study. During year three, we aim to enroll the majority of participants from the active client pool at the Trauma Center and expand to other agencies as necessary (i.e. enrollment targets are not met in a timely manner).

9. **Expected period of study for each individual subject:** Participants will be asked to commit to 12 weeks of twice-weekly 30 minute sessions in addition to assessments at

pre-treatment, mid-treatment, post-treatment, and 1-month follow up. Additionally, these sessions will be administered adjunctive to ongoing individual therapy sessions. Thus, participants will be asked to commit to maintaining ongoing individual therapy above and beyond the neurofeedback sessions. Overall the maximum participation will be approximately 9 months including follow-up.

10. Concise protocol
Rationale for implementation of neurofeedback treatment at the Trauma Center

Researchers have reported an improvement in overall cognitive and emotional functioning, a reduction in symptoms of trauma-related syndromes, and a general improvement in clinical symptoms in both short-term and long-term follow-ups of neurofeedback training (e.g., Peniston and Kulkosky, 1991; Peniston, Marrina, Deming, and Kulkosky,1993). However, there are not sufficient randomized, controlled data to show strong support in these findings.

Neurofeedback offers the field of psychology a relatively new strategy for treating complex, historically treatment resistant disorders. Moreover, there is some evidence that it can be effective in enhancing cognitive performance and modulating arousal for individuals who are not diagnosed with a psychological condition. When considering the long-term risks inherent in current medication treatments (and/or lack of response to more traditional psychological interventions) along with the evidence of the efficacy of neurofeedback, it clarifies the importance of further trials. It is possible that in the future, neurofeedback will not only be an adjunctive treatment, but also perhaps become a primary line of treatment for previously believed "treatment-resistant" conditions. Hirshberg, Chiu, and Frazier (2005) state:

"EEG biofeedback meets the American Academy of Child and Adolescent Psychiatry criteria for 'Clinical Guidelines' for treatment of ADHD, seizure disorders, anxiety (e.g., obsessive-compulsive disorder, GAD, PTSD, phobias), depression, reading disabilities, and addictive disorders. This finding suggests that EEG biofeedback always should be considered as an intervention for these disorders by the clinician" (p. 12).

Complex trauma is a major condition treated at the Trauma Center. The primary domains targeted in treatment interventions for complex trauma are arousal and affect dysregulation. Initial studies have provided evidence for the benefits of neurofeedback not only for ameliorating these primary symptoms, but also symptoms of complex trauma and disorders of extreme stress such as depression, attention deficits, and substance use. Neurofeedback treatment advocates argue it is a potential intervention to help chronically traumatized individuals normalize the brain's response to stress (Scaer, 2007). Additionally, neurofeedback could be a primary mechanism of change in modulating arousal, which represents a benefit to Trauma Center clients by helping to restore equilibrium. Neurofeedback has the potential to offer increased affect regulation, enhanced attention, and enhanced self-regulation.

Preliminary results

Of participants enrolled in our first-year feasibility pilot, we have found a significant reduction in PTSD symptoms ($p < .05$) from baseline to the 1 month assessment as measured by the total score on the Davidson Trauma Scale (DTS). Additionally, there is a significant reduction in avoidance/numbing symptoms on the DTS at 1 and 2 month assessments. Overall reductions in the total score on the DTS were maintained at month 2; however, results did not reach significance due to a lack of power caused by missing data. We have not found significant differences in functioning on other measures, including an overall symptom inventory, and measures of positive and negative affect.

Year 2 represented a more controlled trial. During this time, sensor placement and procedures have been protocolized. Additionally, assessments were administered at standardized points. This permitted more in-depth analyses of change over the course of treatment. We have found a statistically (although not yet clinically) significant drop in total PTSD symptoms as measured by the Davidson Trauma Scale (time 1 = 70.96, time 40 = 54.77; *pr2* = .21). Additionally, we have found significant drops on the Affect Dysregulation subscale of the Inventory of Altered Self-Capacities (time 1 = 24.33, time 40 = 16.82; *pr2* = .56). In an effort to understand more about the mechanisms of neurofeedback, we performed multilevel mediation analyses testing whether PTSD symptom change mediated affect dysregulation or vice-versa. We found that the model with affect dysregulation mediating PTSD symptom change is a much better fit, resulting in a 93% mediation.

Study Design

General Study Design.

The proposed year three pilot study continues to be exploratory in nature; however, we are adding a waitlist control group, reducing the number of sessions, consolidating to a single sensor placement, changing inclusion criteria, and adding assessment measures. Thus, the overall study design will be as follows: 1) potential participants will be screened via a telephone interview. This interview will assess whether participants will meet the initial inclusion criteria for the study. 2) Participants who meet initial eligibility requirements will be scheduled for an in-person assessment. During this meeting, participants will be briefed on the study, study technicians will assess understanding of consent, and the participant will indicate consent by signing the form. 3) Participants will complete baseline assessments. During the baseline assessment appointment, participants will be excluded from the study if they a) do not meet criteria for PTSD based on their CAPS Diagnostic Interview or b) receive a score of 40 or higher on the DES. 4) Participants will be randomly assigned to either waitlist control or active treatment. 5) Participants assigned to active treatment will be scheduled with the study technician for twice weekly appointments. Participants assigned to the waitlist will be given information on completing assessments. 6) At the conclusion of treatment (or waitlist period), participants will complete the termination assessment and receive compensation. 7)

Participants assigned to the waitlist control group will be enrolled in the active treatment condition. The following is an illustration of the design:

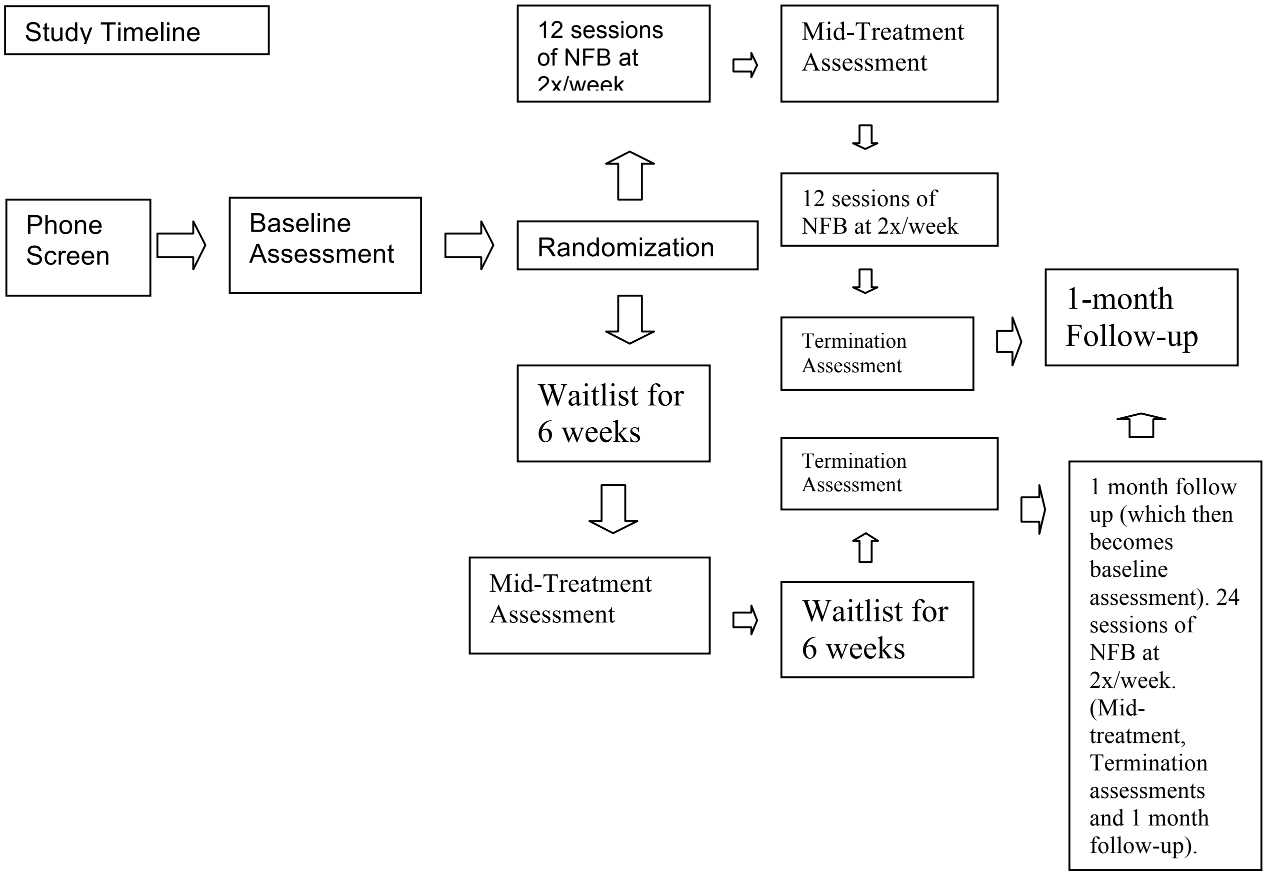

Active Treatment

As stated earlier, we plan to enroll a total of 40 participants, with 20 of those being randomly assigned to receive the active treatment intervention. The training will follow a flexible, principle-based manual that provides rules to adjust the training protocol based on the clinical response (defined as the number of over- or under-arousal symptoms) of each participant. The specifics of this protocol have been refined during our pilot work, and adjustments have been made to maximize efficacy. NFB training will be done using a bipolar placement with T4 as the active site, P4 as the reference site, and the left ear (A1) as the ground (consistent with previous research that demonstrates increased R temporal lobe activation in PTSD [1-3]. We will be employing standard inhibit frequencies and a beginning reward frequency based on previous research [4, 5] and based on experience gained during years 1 and 2 of the current study.

Adjustment of the reward band will be based on how the participant responds between sessions. Participants will complete a short checklist after every session. Adjustments will be made based on rated symptoms of over-arousal (including nightmares; sleep difficulties; hyperactivity; aggressive behavior, anger, anxiety; and self-reports of high

arousal including self-harm, suicidal and/or homicidal ideation), and symptoms of under-arousal (including inattention, decreased alertness or mental clarity; nausea; depressive symptoms; and decreased energy/fatigue). Intervention technicians may also be in regular contact with participants' psychiatrist and/or psychologist.

Participants will have 24 training sessions, twice weekly, each lasting up to 30 minutes. They will be seated comfortably and electrodes applied. Impedance will be measured for each electrode and maintained below 10 kilo ohms. Weekly changes will be captured using feedback from each subject by asking them "do you feel better?". If after the mid-treatment assessment the subject does not improve and has answered that they do not feel better, then protocol will be determined by the QEEG collected at pre-treatment.

Participants will receive auditory and visual feedback indicating reward. Feedback (reinforcement) is provided by means of beeps and simple computer games. During the training, the reward condition is either extended or removed based on the degree to which the amplitude of the measured EEG activity in the targeted spectral bands meets or fails to meet the training parameters or goals. The reinforcement used for operant conditioning is earning more beeps and advancing the computer game. We will use the EEGer neurofeedback system manufactured by EEG Spectrum International.

Control Group

We propose to enroll a cohort of 20 individuals who will provide a *waitlist* control against which to compare the results obtained from the neurofeedback group. The control group will consist of individuals receiving individual psychotherapy services at the Trauma Center or other community agencies. Maintenance of ongoing, weekly psychotherapy will be a requirement for participation. Waitlist participants will be asked to complete the same measures at the same time points as individuals in the active treatment. Overall, participants in the control group will complete a baseline assessment, six interim assessments, and the termination assessment along the same timeline as those in the active treatment condition. Participants for the control will be randomly selected and all waitlist participants will be offered the active treatment at the end of the waitlist period. Once these participants begin receiving neurofeedback, they will follow the same assessment time-line as the original active treatment group by being assessed at mid-treatment, post-treatment and one-month follow up.

Participant Information

Description of the methods to be used to assure informed consent of each participant prior to participation. All adults interested in receiving neurofeedback (NFB) services at the Trauma Center (or other clinics and agencies to which we open inclusion) will be invited to be screened for participation in this study on the role of NFB as a method of affect regulation in the treatment of adults with complex adaptation to chronic interpersonal trauma exposure. Prior to the screening, potential participants will be provided a detailed verbal description of the study. The initial screening will take place

via telephone; therefore, verbal consent to participate in the screening will be obtained. If the potential participant meets inclusion criteria, s/he will be scheduled for an in-person assessment. Upon arrival, potential participants will be provided a written copy of the study consent form for review and consideration. The research coordinator or other designated study technician will explain the consent form and assess individuals' understanding of the contents. Once study personnel are satisfied that the individual understands the consent and the individual agrees to continue, the baseline assessment will be conducted over the course of two meetings. The first baseline assessment meeting will take approximately two hours. The second baseline assessment meeting will include collecting the QEEG which will take approximately two hours. After this baseline assessment, participants will be randomized to either the waitlist control or active treatment group. Availability of NFB services at the Trauma Center will not be contingent upon study participation, and as such study refusal will have no bearing or impact on the provision of NFB or other clinical services at the Trauma Center. In the event that study participants withdraw consent for study participation at any point prior to, during or following study participation, this will have no bearing on their ability to continue to receive NFB or other services at the Trauma Center and their data will be destroyed upon request.

Inclusion Criteria. Eligible individuals will have a positive history of exposure to serial or repeated interpersonal trauma or neglect. Participants will also have a positive history of complex adaptation to their trauma exposure, as evidenced by the CAPS Diagnostic Interview. Participants must have been in stable weekly psychotherapy (within reasonable limits) for at least 3 months before beginning the study and must reasonably be expected to maintain treatment for the duration of participation. Finally, participants will be required to maintain their medication regime for the duration of the study.

Exclusion Criteria. The following study exclusion criteria will be upheld: a score of 40 or higher on the Dissociation Experiences Scale, history of a psychotic or seizure disorder, actively using or having used benzodiazepine medications in the past 6 months, active suicidal ideation or life-threatening self-harm, active substance abuse or dependence disorder requiring detoxification or otherwise compromising ability to engage treatment, blindness or other severe vision impairment prohibiting capacity to engage neurofeedback protocol, mental retardation, and non-English speakers.

Participant Treatment Status and Session Costs. Participation of all study participants will be voluntary and will be provided on an adjunctive basis to individuals enrolled in weekly psychotherapy either at the Trauma Center or other practice. Participants who are not seen at the trauma Center will be required to sign an active bi-directional release of information form with their current primary treating clinician.

Study participants will be provided neurofeedback training as an adjunct to ongoing weekly psychotherapy. Sessions will be provided at no charge.

Treatment Integrity. The Trauma Center has retained a cohort of 6 – 8 clinicians trained in neurofeedback and actively providing training to study participants, as well as a

minimum of two Trauma Center senior supervisors trained in neurofeedback who are actively involved in implementation of neurofeedback in their private, trauma-focused practices, and who are available to project staff for ongoing technical assistance and supervision. The study technician will be required to attend a weekly supervision meeting. The primary function of supervision will be to insure fidelity to treatment protocols, and identify and remediate any adverse responses. The second function will be to insure consistent administration, scheduling and collection of client baseline, mid-treatment, weekly and outcome data. The research coordinator will review at least 25% of NFB training computer and paper files in order to insure treatment fidelity.

Assessment Measures

Psychological, behavioral, and neuropsychological areas of functioning will be assessed at the following time points: 1. pre-treatment; 2.mid-treatment; 3. post-treatment; and 4. one-month follow-up. We will use the following measures:

I. Client History

1. Demographics and Treatment History. Administration: 5 minutes at time point 1.

2. Traumatic Events Screening Inventory (TESI). The TESI is a clinician administered questionnaire to assess traumatic events that have occurred in adults. Administration: 15 minutes at time point 1.

II. Clinical Assessments

1. Clinician Administered PTSD Scale (CAPS, [8]). The CAPS is considered the gold standard for the assessment of PTSD (National Center for PTSD Research). It is a clinician administered 30-item interview that corresponds to DSM-IV criteria for PTSD. Each item of the CAPS has two parts, frequency and intensity, which are both scored on a 5-point scale from 0 to 4. A general cut-off rule of frequency greater than or equal to 1 and intensity greater than or equal to 2 for a symptom to count towards diagnosis will be employed in assigning PTSD diagnosis. Administration: 60 minutes at time points 1, 3, and 4.

2. The Davidson Trauma Scale (DTS, [9]). This is a well validated self-report measure of PTSD with clinical reference norms for adults. Administration: 10 minutes at time points 1, 2, 3, and 4.

3. Inventory of Altered Self-Capacities (IASC, [14]).The IASC is a 63-item standardized measure of disturbed functioning in relation to self and others. The IASC measures seven domains of functioning: Interpersonal Conflicts, Idealization-Disillusionment, Abandonment Concerns, Identity Impairment, Susceptibility to Influence, Affect Dysregulation, and Tension Reduction Activities. Administration: 10 minutes at time points 1, 2, 3, and 4.

4. Behavior Rating Inventory of Executive Functioning for Adults (BRIEF A, [15]) The BRIEF is a 75-item standardized measure of the following areas of executive functioning: inhibit, self-monitor, plan/organize, shift, initiate, task motor, emotional control, working memory, and organization of materials. Administration: 10 minutes at time points 1, 2, 3, and 4.

5. Dissociative Experiences Scale (DES, 26). The DES contains 28 items and measures the frequency of dissociative experiences, from 0% = never to 100% = always, on an 11-point scale. The coefficient alphas for internal consistency ranged from .83 to .93, and the test-retest reliability was .79, with a 6-8-week test-retest interval; reliability and validity of the scale are well-documented. We will exclude subjects with high dissociation, i.e., scores >25. Administration: 10 minutes at time points 1, 2, 3, and 4.

6. CNS Questionnaire. The CNS Functioning Assessment is designed to assess functioning in a number of areas: the sensory, emotional, clarity/cognitive, energy, memory, movement, pain, and miscellaneous areas. Administration: 10 minutes at time points 1 and 3.

III. Neuropsychological measures

We chose several neuropsychological measures that tap areas that have been shown to be impaired in PTSD and that have been shown to improve after NF training:

1. Quantitative EEG (Q EEG). The EEG Spectrum System using a 19-point placement of EEG sensors on the scalp will be used to measure overall EEG activity of the brain. Administration: 2 hours at time points 1 and 4.
2. WebNeuro. This computerized battery measures the traditional cognitive domains of emotion, thinking, feeling and self-regulation to best capture that individual's overall functional wellness. Administration: 30 minutes at time points 1 and 4.

Chart of Time Line of Assessments: This is chart describes the timeline of when assessments will be collected.

Time 1: Baseline	Time 2: Mid-Treatment	Time 3: Post-Treatment	Time 4: Follow-up (1month)
CAPS (sx at 1 wk + 1 month)		CAPS (sx at 1 wk)	CAPS (sx at 1 wk and at one month)
TESI			
DTS	DTS	DTS	DTS
IASC	IASC	IASC	IASC
DES		DES	DES
BREIF A	BREIF A	BREIF A	BREIF A
CNS		CNS	
QEEG		QEEG	
WebNeuro		WebNeuro	

Data Analysis Plan

Multilevel regression (i.e., mixed-effects regression, random-coefficients modeling, hierarchical linear modeling) will be used for all analyses [18]. Strengths of this approach include (a) capability of handling missing data and unbalanced designs (i.e., the number of assessment points and the timing of assessments can vary across subjects), (b) very efficient and powerful estimation procedures that utilize all data points available, and (d) modeling flexibility that provides multiple options for how to model time and allows for the inclusion of continuous or categorical, time invariant or time varying, predictors and covariates. This modeling flexibility enables the sophisticated examination of the

relationship among multiple variables over time, the type of analyses that is necessary to elucidate mechanisms of change during the course of treatment.

The modeling flexibility and power of the multilevel regression approach makes it very well suited to test all of the study's hypotheses. To test PTSD symptom change, two different types of analyses will be conducted. First, the administration of the DTS on several occasions from the baseline assessment, during treatment, and throughout the follow-up period will allow us to capitalize on the advantages of contemporary growth curve approaches that produce very efficient estimates of change over time and increased power to detect differences across treatment conditions (as power is a function of both sample size and number of assessments). Therefore, growth curve analyses will be conducted from a multilevel regression framework to evaluate change over time in PTSD symptoms as assessed by the DTS and how change over time differs as a function of treatment condition [19].

To supplement these analyses and document group differences in change over time on the CAPS, which will only be administered on four occasions (pre-, mid-, post-treatment, and follow-up), analyses with time modeled as a categorical variable (via a set of dummy coded variables) will be conducted. This approach is analogous to conducting a repeated measures ANOVA that capitalizes on the numerous benefits of the multilevel regression approach.

To test changes in affect dysregulation and mediation models, we will use the same growth curve approach used to evaluate PTSD symptom change. Data that was collected several times throughout the protocol (e.g., DTS, affect dysregulation subscale of the IASC, and the BRIEF measure of executive functioning) will be submitted to a series of multi-level meditational analyses [20, 21]. This approach is increasingly being applied to data from randomized control trials to identify mechanisms of change and to test whether the proposed mediation model differs across active treatment and control conditions (e.g., [22-24]). This analytic strategy examines the trajectory of an outcome variable with and without the proposed mediator included as a time-varying covariate. For instance, a substantial reduction in the regression coefficient indicating the degree to which PTSD changes over time when the affect dysregulation subscale of the IASC is added as a time varying covariate and a significant indirect from time (which is the predictor variable in growth curve analyses and can be included the model in a number of ways including session number of amount of time elapsed since the baseline assessment) to PTSD through affect dysregulation (tested using the distribution of products test; [25]) would support the hypothesis that reductions in PTSD due to NF training are accounted for by reductions in affect dysregulation. The meditational analyses will first be conducted on data from the active treatment group only. Then moderated-mediation analyses will be conducted to evaluate whether the proposed meditational model holds up for the active treatment condition but not the waitlist control condition, which would provide the most rigorous test of the proposed meditational model (e.g., [22]).

Potential risks: Participants may experience mild side effects after neurofeedback training, such as headaches, difficulty falling asleep, feeling tired, spacey, anxious, and

agitated or irritable. However, these side effects usually remit within a day after the training session. In order to reduce the likelihood of adverse side effects, participants' experiences will be closely monitored so that frequencies can be adjusted to avoid potential uncomfortable sensations or feelings after treatment sessions.   .

Follow-up of an adverse event or substantial decrease in functioning, even after the date of therapy discontinuation, will continue until the event or its sequelae resolve or stabilize at a level acceptable to the investigator. This follow-up of adverse events will include assessment by the principal investigator of the participant's need for immediate and ongoing treatment, including crisis intervention, pharmacological treatment, individual psychotherapy, Yoga, or group psychotherapy. If appropriate, the participant will be offered these services through the Trauma Center. If the participant requests, Dr. van der Kolk will help arrange for continued care by the individual's own physician and/or therapist, or make a referral to another practitioner or facility as it may be appropriate.

Potential benefits: Participants may experience and improvement in overall cognitive and emotional functioning, a reduction in symptoms of trauma-related disorders, and a general improvement in clinical symptoms. Initial studies have supported the benefits of neurofeedback in the domains of arousal, affect dysregulation, depression, attention deficits, and substance use.

Remuneration or other awards to participants: Participants will be compensated for completing the baseline, mid-treatment, termination, and follow-up. Participants randomized to the active treatment group will be paid $50 for pre- and mid-treatment assessments, $70 for the post-treatment assessment, and $80 for the 1-month follow-up. Completers of all 4 evaluations of the active treatment group will receive a total of $250. Participants randomized to the waitlist control for 4 months, then have the option of receiving neurofeedback will be compensated $50 for pre- and mid-treatment assessments, $70 for the post-treatment assessment, and $80 for the 1-month follow-up, then $50 for mid-treatment assessments, $70 for the post-treatment assessments and $80 for the 1-month follow-up. Completers of the waitlist control group who then receive neurofeedback treatment will receive a total of $450.

Procedures for protecting participants' confidentiality: For study purposes, all data will be de-identified and a numeric coding system and a numeric coding system has been put in place. All data entered into study databases are identified by this numeric system only.

Name of funding/granting agency, and annual and total budget amount: Total initial 3-year funding award =$493,760. Total expended in Y01= $140,046. Total to be expended in Y02 ~ $160,000. Estimated remaining balance to conduct Y03 project ~ $193,000. Foundation has verbally agreed to increase amount and duration of final year of award as necessary to ensure successful conduct of project as delineated above.

Anticipated final product: The anticipated final products for this evaluation will include the following: 1) most importantly we aim to show proof-of-concept by demonstrating a

statistically and clinically significant improvement in reported symptoms of participants which will be suitable for publication in scholarly journals, 2) delineation of specific protocols, guidelines, sequences and/or clinical decision-making strategies regarding application of NFB with complexly traumatized adults; 3) compilation of written narrative case studies to illustrate NFB adaptation process and provide descriptive outcomes derived from NFB application; and 4) grant applications for further controlled trials of NFB.

Signature of Principal Investigator:

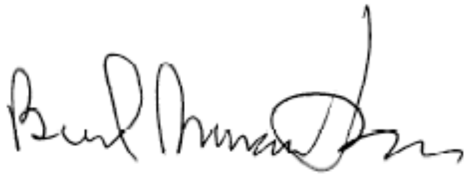

Citations

1. Rauch, S.L., et al., *A Symptom Provocation Study of Posttraumatic Stress Disorder Using Positron Emission Tomography and Script-Driven Imagery.* Archives of General Psychiatry, 1996. **53**: p. 380-387.
2. Lanius, R.A., et al., *Brain activation during script-driven imagery induced dissociative responses in PTSD: A functional magnetic resonance imaging investigation.* Biological Psychiatry, 2002. **52**(4): p. 305-311.
3. Georgopoulos, A.P., Tan, H-R.M., Lewis, S.M., Leuthold, A.C., Winskowski, A.M., Lynch, J.K. et al., *The synchronous neural interactions test as a functional neuromarker for post-traumatic stress disorder (PTSD): a robust classification method based on the bootstrap* Journal of Neural Engineering, 2010. **7**(1): p. 1-7.
4. Begic D, H.L., Jokic-Begic N, , *Electroencephalographic comparison of veterans with combat-related post-traumatic stress disorder and healthy subjects.* Int J Psychophysiology, 2001. **40**(167–172).
5. Jokic-Begic, N.B., D., *Quantitative electroencephalogram (qEEG) in combat veterans with post-traumatic stress disorder (PTSD).* Nord J Psychiatry 2003. **57**: p. 351–355.
6. Thompson, L., Thompson, M. & Reid, A., *Neurofeedback outcomes in clients with Asperger's Syndrome.* Applied Psychophysiology and Biofeedback, 2010. **35**(1): p. 63-81.
7. Goodman, L., Corcoran, C., Turner, K., Yuan, N., & Green, B., *Assessing traumatic event exposure: General issues and preliminary findings for the Stressful Life Events Screening Questionnaire.* Journal of Traumatic Stress, 1998. **11**(3): p. 521-542.
8. Blake, D.D., et al., *The development of a clinician-administered PTSD scale.* Journal of Traumatic Stress`, 1995. **8**(1): p. 75-90.
9. Davidson, J.R.T., H.M. Tharwani, and K.M. Connor, *Davidson Trauma Scale (DTS): Normative scores in the general population and effect sizes in placebo-controlled SSRI trials.* Depression and Anxiety, 2002. **15**: p. 75-78.

10. First, M.B., Spitzer, Robert L, Gibbon Miriam, and Williams, Janet B.W, *Structured Clinical Interview for DSM-IV-TR Axis I Disorders, Research Version, Patient Edition. (SCID-I/P)*. 2002, New York: Biometrics Research, New York State Psychiatric Institute.
11. Derogatis, L.R., *Brief Symptom Inventory*. 1975, Baltimore: Clinical Psychometric research.
12. Derogatis, L.R., *The SCL-R-90 Manual I: Scoring, Administration and Procedures for the SCL-90*. 1977, Baltimore: Clinical Psychometric research.
13. Endicott J, N.J., Harrison W, Blumenthal R., *Quality of Life Enjoyment and Satisfaction Questionnaire: a new measure.* Psychopharmacol bull 1993. **29**(2): p. 321-326.
14. Briere, J.R., M., *The Inventory of altered self-capacities: A standardized measure of identity, affect regualtion, and relationship disturbance.* Assessment, 2002. **9**(3): p. 230-239.
15. R.M. Roth, P.K.I.G.A.G., *Behavioral Rating Inventory of Executive Function—Adult version*. 2005, Lutz, FL: Psychological Assessment Resources, Inc.
16. Connors, C.K., *Conners' Continuous Performance Test computer program user's guide*. 1992, Toronto, Ontario: Multi-Health Systems.
17. Rey, A., *L'examen clinique en psychologie [The clinical examination in psychology]*. 1964, Paris: Presses Universitaires de France.
18. Singer, J.D.W., J.B., *Applied Longitudinal Data Analysis: Modeling Change and Event Occurrence*. 2003, Oxford: Oxford University Press.
19. Raudenbush, S.W., *Comparing personal trajectories and drawing causal inferences from longitudinal data.* Annual Review of Psychology, 2001. **52**: p. 501-525.
20. Bauer, D., Preacher, K., & Gil, K. , *Conceptualizing and testing random indirect effects and moderated mediation in multilevel models: New procedures and recommendations.* . Psychological Methods, 2006. **11**: p. 142-163.
21. Kenny, D.A., Korchmaros, J. D., & Bolger, N. , *Lower level mediation in multilevel models.* Psychological Methods, 2003. **8**: p. 115-128.
22. Hofmann, S.G., Meuret, A. E., Rosenfield, D., Suvak, M. K., Barlow, D. H., Gorman, J. M., Shear, M. K., & Woods, S. W., *Preliminary Evidence for Cognitive Mediation during Cognitive Behavioral Therapy of Panic Disorder.* Journal of Consulting and Clinical Psychology, 2007. **75**: p. 374-379.
23. Moscovitch, D.A., Hofmann, S. G., Suvak, M., & In-Albon, T. , *Mediation of changes in anxiety and depression during treatment for social phobia.* Journal of Consulting and Clinical Psychology, 2005. **75**: p. 945-952.
24. Smits, J.A., Rosenfield, D., McDonald, R., & Telch, M. J., *Cognitive mechanisms of social anxiety reduction: an examination of specificity and temporality.* Journal of Consulting and Clinical Psychology, 2006. **74**: p. 1203-1212.
25. MacKinnon DP, L.C., Williams J. , *Confidence intervals for the indirect effect: distribution of the product and resampling methods.* Multivariate Behavioral Research, 2004. **39**: p. 99-128.
26. Bernstein EM, Putnam F. Development, Reliability, and Validity of a Dissociation Scale. J Nerv Ment Dis 1986. **174**: 727-735.
